# Supplementary material for: Causal interplay between lactose intolerance and gut microbiota: a combined bidirectional Mendelian randomization and in vivo validation study
Source: Front Nutr. 2026 Jun 1;13:1803337. doi: 10.3389/fnut.2026.1803337 (PMC13265576; doi:10.3389/fnut.2026.1803337)
Supplement: Supplementary file 1 [file Data_Sheet_1.zip › supplementary materials/Forward/leave one out cross validation/ebi-a-GCST90027621.finngen_R12_E4_LACTONAS.pdf]

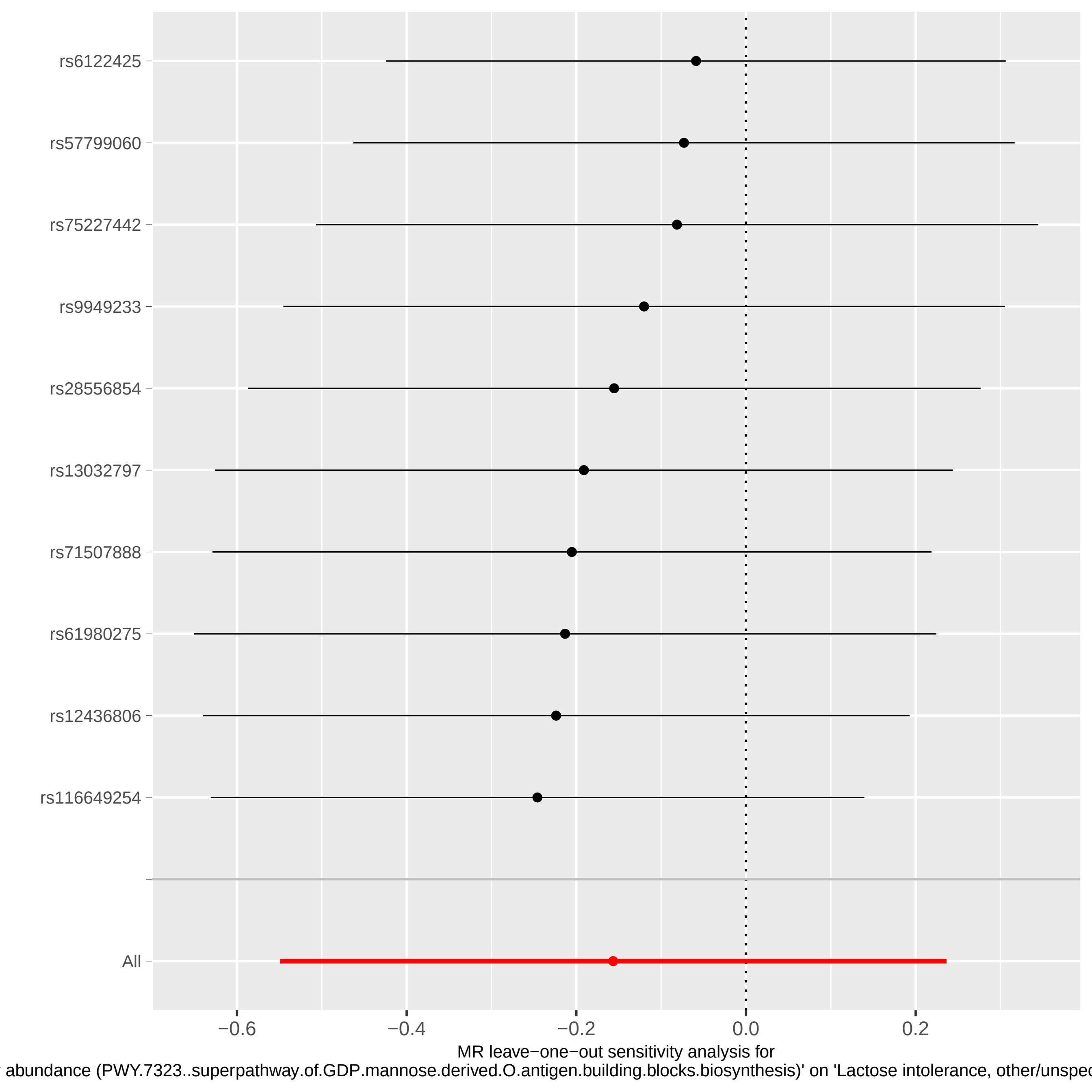
rs6122425
rs57799060
rs75227442
rs9949233
rs28556854
rs13032797
rs71507888
rs61980275
rs12436806
rs116649254
All
−0.6
−0.4
−0.2
0.0
0.2
MR leave−one−out sensitivity analysis for
abundance (PWY.7323..superpathway.of.GDP.mannose.derived.O.antigen.building.blocks.biosynthesis)' on 'Lactose intolerance, other/unspe
